# The role of Vitamin D supplementation in cardiovascular health: A reappraisal

**DOI:** 10.21542/gcsp.2025.10

**Published:** 2025-02-28

**Authors:** Shrishti P. Khetan, Shruti Suresh Suvarna

**Affiliations:** American University of Barbados, Bridgetown, Saint Michael, Barbados

**To the Editor,**

The role of vitamin D supplementation in cardiovascular health sparks ongoing debate and remains inconclusive. Vitamin D deficiency has been implicated in the development of cardiovascular diseases (CVD), including myocardial infarction and stroke^1^, however, the efficacy of supplementation to reduce the risk of these outcomes has recently been questioned. This letter is an attempt to reevaluate the literature and present clinicians with the applicable evidence-based recommendations.

Recent meta-analysis and clinical trial studies are inconclusive and showing mixed results. Though observational studies observed an inverse relation between vitamin D levels and risk of CVD, intervention trials, particularly those involving supplementation, show minimal outcomes for CVD. However, clinical trials like the VITAL study performed on a large population failed to demonstrate any decrease in the risk of myocardial infarction or stroke by taking vitamin D supplements. These findings underscore the importance of developing a more sophisticated understanding of supplementation in the context of cardiovascular health.

Possible ways through which vitamin D might prevent cardiovascular diseases have been hypothesized. vitamin D is postulated to affect blood pressure through the renin-angiotensin-aldosterone system, bring anti-inflammatory effects and negatively influence the progress of atherosclerosis, as illustrated in Figure 1. However, the clinical relevance of these mechanisms is not supported by recent studies. They are still considered biologically reasonable, although their conversion to tangible cardiovascular improvements has been unsatisfactory.

**Figure 1.**
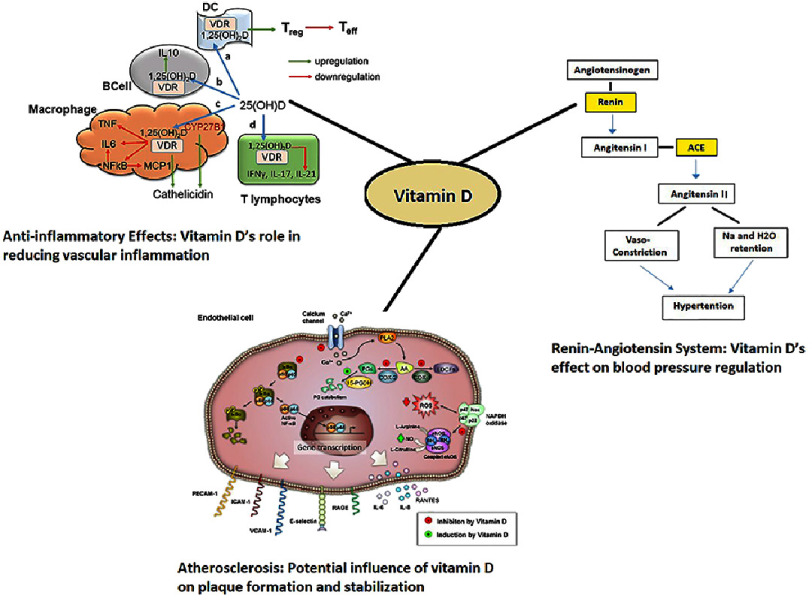
Proposed mechanisms for Vitamin D’s role in cardiovascular health.

However, some populations may still receive benefit. Severe vitamin D deficiency (<10 ng/mL) has been implicated in high risk of cardiovascular events. Osteoporosis or a history of fractures, and ethnicity (darker-skinned populations) increases the risk of CVD^2^. For these groups, supplementation could fill the deficit and possibly reduce cardiovascular risk. Tailored and personalized intervention programs should focus on these high-risk groups rather than continue supporting supplementation for everyone.

Thus, scientific evidence based on recent literature does not warrant use of Vitamin D supplementation for the primary maintenance and prevention of cardiovascular diseases. Clinicians ought to intervene in achieving desirable vitamin D status in a range of 20–30 ng/mL because of its influence on bone health with possible secondary cardiovascular outcomes. For high-risk groups, deficiencies should be screened, and supplementation should be used specifically to correct a deficiency, rather than applied universally.

Overall, vitamin D supplementation appears to have little effect on cardiovascular endpoints and further work is needed to establish the role of vitamin D in cardiovascular health. Clinicians should take an individualized, evidenced based approach to deficiency correction and avoid needless supplementation. There are gaps in current research that future studies ought to address, such as long-term effect of supplementation, and its impact on special high-risk groups.

## References

[1] Latic N, Erben RG (2020). Vitamin D and cardiovascular disease, with emphasis on hypertension, atherosclerosis, and heart failure. Int J Mol Sci.

[2] Oliver SL, Santana KV, Ribeiro H (2022). The effect of sunlight exposure on vitamin D status in countries of low and high latitudes: A systematic literature review. Curr Nutr Rep.

[3] Ruiz-García A, Pallarés-Carratalá V, Turégano-Yedro M, Torres F, Sapena V, Martin-Gorgojo A, Martin-Moreno JM (2023). Vitamin D supplementation and its impact on mortality and cardiovascular outcomes: Systematic review and meta-analysis of 80 randomized clinical trials. Nutrients.

[4] Pei Y, Zhang Y, Peng X, Liu Z, Xu P, Fang F (2022). Association of vitamin D supplementation with cardiovascular events: A systematic review and meta-analysis. Nutrients.

[5] Manson JE, Bassuk SS, Buring JE (2020). Principal results of the vitamin D and omega-3 trial (VITAL) and updated meta-analyses of relevant vitamin D trials. J Steroid Biochem Mol Biol.

